# Factors that preclude the conclusion of a kidney transplant protocol from a living donor

**DOI:** 10.1590/2175-8239-JBN-2024-0157en

**Published:** 2026-04-17

**Authors:** Alfonso Rodríguez Ojeda, Raúl Edgar Santacruz Adi, Nelly Gonzalez Audiffred, Rogelio Iván Silva Rueda, Fabiola Pazos Pérez, Maria Inés Gil Arredondo, Javier Rivera Flores, Jorge Velez Silva, Adriana Flores Palacios, Juan Carlos H. Hernández Rivera

**Affiliations:** 1 Instituto Mexicano del Seguro Social, Centro Médico Nacional Siglo XXI, Hospital de Especialidades, Servicio de Nefrología, Ciudad de México, México.; 2 Instituto Mexicano del Seguro Social, Centro Médico Nacional Siglo XXI, Hospital de Especialidades, Unidad de Investigación Médica en Enfermedades Nefrológicas, Ciudad de México, México.

**Keywords:** Transplant Protocol, Living Donors, Graft Rejection

## Abstract

**Introduction:**

A kidney transplant (KT) is a renal replacement therapy that offers greater survival and quality of life; however, it is not always successfully completed due to social, physical, medical, immunological factors, or lack of adherence.

**Methods:**

This retrospective, analytical, longitudinal study included patients in the KT protocol from a living donor between May 2021 and November 2022. Factors preventing completion were evaluated. Comparative analyses and cumulative incidence curves were used to assess time to protocol completion or discontinuation. The analysis was performed using IBM SPSS Statistics, version 26 (IBM Corp., USA).

**Results:**

Among 240 donor–recipient pairs, 116 (48.3%) successfully completed KT, whereas 124 (51.6%) did not. Reasons for non-completion included medical causes (59/124; 47.6%), immunological incompatibility (28/124; 22.6%), psychosocial factors (14/124; 11.3%), and other causes (23/124; 18.5%). The median time to protocol acceptance or discontinuation was 151 days (IQR 115–186). Among individuals who ultimately underwent KT, the median completion time was also 151 days (IQR 109–231).

**Conclusions:**

Medical conditions were the predominant barriers to completing living-donor KT protocols, followed by immunologic and psychosocial factors. Streamlining medical assessments, implementing early immunologic screening, and reinforcing psychosocial support may enhance protocol completion rates.

## Introduction

An estimated 8–15% of the global population is affected by chronic kidney disease (CKD). In middle-income countries, noncommunicable chronic diseases account for approximately 71% of the total disease burden, and end-stage renal disease (ESRD) represents about 15% of annual health expenditure^1,2^.

Not all patients with CKD are candidates for kidney transplantation (KT). Compared with deceased-donor transplantation, living-donor KT confers several advantages, including superior patient and graft survival and a shorter time to transplant^3,4,5,6^. Patients receiving hemodialysis (HD) or peritoneal dialysis (PD) commonly experience progressive deterioration in overall health, employment status, emotional well-being, energy, sexual function, and sleep^7,8^. By 2022 in Mexico, nearly 7 of every 10 KTs were from living donors. Low transplant rates in low- and middle-income countries are primarily attributable to deficiencies in health infrastructure. Transplant outcomes, particularly medium- and long-term survival depend on access to immunosuppressive medications, nutritional status, and control of infectious diseases. Social, cultural, and economic inequities, together with multiple immunologic and nonimmunologic factors, influence both access to transplantation and post-transplant outcomes^9^.

Timeliness in living-donor evaluation is critical, with objectives that include reducing comorbidity and lowering costs for patients on renal replacement therapy. Review articles report variability in donor evaluation timelines: target intervals of approximately 18 weeks have been described in the United States and the United Kingdom^10,11,12^. In a Canadian cohort of 887 donors, the median time from referral to donation was 10.5 months (IQR 6.9–17.7 months); notably, 122 of 360 patients awaiting transplantation had to initiate renal replacement therapy because of delays^13^. A U.S. study reported a median time of 9.2 months, with African American donors experiencing a slightly longer median interval (10.2 months) than non–African American donors (8.9 months). The evaluation process is commonly divided into five stages^1^: referral^2^, medical screening^3^, evaluation^4^, clearance, and^5^ donation^14^. A systematic review suggests a typical median timeline of approximately 10 months; up to 25% of protocols exceed 16 months, often because of additional investigations, required weight loss, or substance-abstinence interventions that can add 3–9 months^6,15,16^.

Multiple factors may preclude completion of living-donor KT; these deliberations prioritize the safety of both donor and recipient^17^. This study aimed to identify factors associated with non-completion of living donor kidney transplant protocols, as well as the average times of each operationally designated group.

## Methods

This was a retrospective, analytical longitudinal study conducted between May 1, 2021, and November 30, 2022. All available cases were analyzed. The study included patients with CKD stage 4–5 KDIGO (Kidney Disease: Improving Global Outcomes) who were sent as donor-recipient pairs, of any gender, and older than 18 years. Exclusion was considered for those lacking required epidemiological data in their clinical records; however, no recipient-donor pair was ultimately excluded. The study was approved by the IMSS Local Committee for Research and Ethics in Health, approval number 3601, with institutional registration number R-2023-3601-050, dated February 3, 2023.

The data for each donor-recipient pair, including assessments from each specialist involved (cardiologist, urologist, maxillofacial surgeon, nephrologist, psychiatrist, nutritionist, internal medicine specialist, etc.), as well as biochemical and office studies, were reviewed in the institutional electronic and physical records with prior authorization from the ethics and research committee.

The reasons for not completing the protocol and thus not reaching transplantation were operationally grouped into four categories: 1) Medical: arterial hypertension, proteinuria, lithiasis, renal atrophy, arterial stenosis, polycystic disease, malignancy or suspicion of malignancy, ectasia, active infections, cardiac alterations, morbid obesity, urinary sediment abnormalities, metabolic syndrome, diabetes mellitus, etc.; 2) Psychosocial: as determined by psychiatry, such as dementia, or by psychology, such as lack of an adequate family support network; 3) Immunological: persistently positive crossmatch by various methods, including CDC (Complement Dependent Cytotoxicity), highly sensitized antibody reactive panel, or presence of donor-specific antibodies at high titers; and 4) Other: for the purposes of this investigation, “other” included causes that cannot be classified as medical, psychosocial (as determined by a psychiatrist or psychologist), or histocompatibility-related. Examples include donor hesitancy to donate a kidney, recipient decision to discontinue the transplant protocol, recipient death, change of residence to another state or region, or loss of social security.

For quantitative variables, the mean and standard deviation or the median and interquartile range (IQR 25–75) were reported according to the distribution. Categorical variables were described using frequencies and percentages. Group comparisons were made between patients who successfully completed their kidney transplant protocol and those who did not. Student’s t-test was used to analyze differences in means, the Mann-Whitney U test for medians, and Fisher’s exact test or Pearson’s chi-squared test for qualitative variables, as appropriate.

Cumulative incidence curves were constructed from the start of the protocol and concluded when transplants were performed or when the protocol was not completed for any reason. For all statistical analyses described, a p-value < 0.05 was considered statistically significant. All statistical analyses were performed using IBM SPSS Statistics, version 26 (IBM Corp., Armonk, NY, USA).

## Results

Two hundred forty donor-recipient pairs for transplant purposes were included. The potential recipients were men in 126 cases (52.5%) and women in 114 cases (47.5%), without statistical differences. For the potential donor variable, the glomerular filtration rate showed statistical significance with a U-Mann-Whitney p-value of 0.002 (Table 1).

**Table 1:** Baseline characteristics of potential receptors and potential donors prior to the protocol

Variable	Total (n = 240)	Concluded protocol (n = 116)	Did not conclude protocol (n = 124)	p
Potential kidney receptor variables				
Sex				0.115
Men	126 (52.5)	67 (57.8)	59 (47.6)	
Women	114 (47.5)	49 (42.2)	65 (52.4)	
Age (years)*	33 (28–42)	33 (28–39)	34 (27–48)	0.012
Chronic diseases				0.605
DM 1	8 (3.3)	4 (3.4)	4 (3.2)	
DM 2	2 (0.8)	0 (0.0)	2 (1.6)	
SAH	190 (79.2)	93 (80.2)	97 (78.2)	
DM-SAH	28 (11.7)	12 (10.3)	16 (12.9)	
Blood type				0.112
O	166 (69.2)	82 (70.7)	84 (67.7)	
A	60 (25.0)	24 (20.7)	36 (29.0)	
B	11 (4.6)	7 (6.0)	4 (3.2)	
AB	3 (1.3)	3 (2.6)	0 (0.0)	
RRT mode prior KT				0.229
Hemodialysis	100 (41.7)	42 (36.2)	58 (46.8)	
Peritoneal dialysis	100 (41.7)	55 (47.4)	45 (36.3)	
KT	1 (0.4)	0 (0.0)	1 (0.8)	
Anticipated	39 (16.3)	19 (16.4)	20 (16.1)	
CKD etiology				0.26
Undetermined	158 (65.8)	80 (69.6)	78 (62.9)	
DM	24 (10.0)	10 (8.7)	14 (11.3)	
ADPKD	14 (5.8)	5 (4.3)	9 (7.3)	
Focal and segmental GS	8 (3.3)	6 (5.2)	2 (1.6)	
Vesicoureteral reflux	7 (2.9)	5 (4.3)	2 (1.6)	
Lupus nephritis	7 (2.9)	4 (3.5)	3 (2.4)	
GS MP	4 (1.7)	0 (0.0)	4 (3.2)	
SAH	3 (1.3)	2 (1.7)	1 (0.8)	
ANCA Vasculitis	3 (1.3)	0 (0.0)	3 (2.4)	
Nephropathy for Ig A	2 (0.8)	1 (0.9)	1 (0.8)	
Tubulointerstitial nephritis	2 (0.8)	0 (0.0)	2 (1.6)	
Hyperuricemia	2 (0.8)	1 (0.9)	1 (0.8)	
Nephrolithiasis	1 (0.4)	0 (0.0)	1 (0.8)	
Nephroangiosclerosis	1 (0.4)	0 (0.0)	1 (0.8)	
Takayasu arteritis	1 (0.4)	0 (0.0)	1 (0.8)	
Neurogenic bladder	1 (0.4)	1 (0.9)	0 (0.0)	
Radiation nephropathy	1 (0.4)	0 (0.0)	1 (0.8)	
Kidney cancer	1 (0.4)	0 (0.0)	1 (0.8)	
Potential kidney donor variables				
Sex				0.115
Men	93 (38.8)	39 (33.6)	54 (43.5)	
Women	147 (61.3)	77 (66.4)	70 (56.5)	
Age (years)*	35 (28–46)	35 (30–44)	36 (27–48)	0.641
Creatinine (mg/dL)*	0.77 (0.70–0.80)	0.77 (0.70–0.85)	0.78 (0.67–0.87)	0.111
GFR (mL/min/1.72 m^2^ SC)*	109.0 (99.4–119.7)	109.0 (99.4–119.7)	108.1 (88.3–125.6)	0.002
Blood type				0.617
O	188 (78.3)	92 (79.3)	96 (77.4)	
A	42 (17.5)	18 (15.5)	24 (19.4)	
B	9 (3.8)	5 (4.3)	4 (3.2)	
AB	1 (0.4)	1 (0.9)	0 (0.0)	
Relationship				0.346
Cousin	12 (5)	5 (4.3)	7 (5.6)	
Mother/father	42 (17.5)	19 (16.4)	23 (18.5)	
Sibling	86 (35.8)	45 (38.8)	41 (33.1)	
Husband/wife	45 (18.8)	27 (23.3)	18 (14.5)	
Son/daughter	19 (7.9)	4 (3.4)	15 (12.1)	
Brother/sister in law	3 (1.3)	1 (0.9)	2 (1.6)	
Uncle/Aunt	9 (3.8)	4 (3.4)	5 (4.0)	
Grandmother/grandfather	1 (0.4)	0 (0.0)	1 (0.9)	
Niece/Nephew	2 (0.8)	1 (0.9)	1 (0.8)	
None	21 (8.8)	10 (8.6)	11 (8.9)	

Abbreviations – n: number; DM1: diabetes mellitus 1; DT2: diabetes mellitus 2; SAH: Systemic Arterial Hypertension; RRT: Renal Replacement Therapy; KT: Kidney transplant; CKD: Chronic Kidney Disease; ADPKD: Autosomal dominant polycystic kidney disease; GS: Glomeruloesclerosis; MP: Membranoproliferative; ANCA: Antineutrophil Cytoplasmic Antibodies; Ig: Immunoglobulins; mg: milligrams; min: minute; ml: milliliter; m: meter; BSA: Body Surface Area.

Notes – (*) Data expressed as median and 25–75 interquartile range of, group comparison were performed wuth U-Mann-Whitney.

Among the protocol characteristics, some statistically significant variables were observed in potential kidney recipients. The cross-matching test was positive in 15 cases (6.3%), where all cases completed the protocol (chi-square p < 0.001). Panel Reactive Antibody (PRA) class I greater than 30% was found in 24 cases (10%): 8 cases (7.8%) among those who completed the protocol and 16 cases (30.8%) among those who did not, with a statistically significant difference between groups (chi-square p < 0.001). For variables related to potential donors, statistically significant differences between groups (chi-square p < 0.001) were found for proteinuria greater than 300 mg/dL, microscopic hematuria, clinical or office- detected alterations during the protocol, psychological disorders in 4 cases, and infections during the protocol. For kidney function, the glomerular filtration rate was analyzed in 3 groups, showing a significant difference between groups (chi-square p = 0.005) (Table 2).

**Table 2:** Potential receptor and potential kidney donor characteristics during protocol

Variable	Total (n = 240)	Concluded protocol (n = 116)	Did not conclude protocol (n = 124)	p
Potential kidney receptor variables				
Positive cross matching test	15 (6.3)	0 (0.0)	15 (78.9)	<0.001
Panel Reactive Antibody				
Class I (more than 30%)	24 (10.0)	8 (7.6)	16 (30.8)	<0.001
Class II (more than 30%)	22 (9.2)	13 (12.4)	9 (17.3)	0.403
Donor-specific antibody	97 (40.4)	59 (53.6)	38 (64.4)	0.177
Active infections	11 (4.9)	3 (2.6)	8 (7.3)	0.105
Renal abscess	1 (0.4)	0 (0.0)	1 (0.9)	
Peritonitis	1 (0.4)	0 (0.0)	1 (0.9)	
Pneumonia	2 (0.8)	1 (0.9)	1 (0.9)	
HIV	1 (0.4)	0 (0.0)	1 (0.9)	
Latent Tuberculosis	6 (2.5)	2 (1.7)	4 (3.6)	
Anemia	190 (79.2)	96 (82.8)	94 (75.8)	0.185
Psychological disorder	1 (0.4)	0 (0.0)	1 (0.9)	0.297
Abnormal transthoracic echocardiogram	171 (71.3)	93 (80.9)	78 (74.3)	0.241
Abnormalcardiovascular stress test	34 (14.2)	14 (15.1)	20 (25.6)	0.084
Potential kidney donor variables				
Bodyweight alterations				0.234
Underweight	55 (24.4)	29 (25.7)	26 (23.2)	
Overweight	124 (55.1)	65 (57.5)	59 (52.7)	
Obesity class 1	40 (17.8)	16 (14.2)	24 (21.4)	
Obesity class 2	1 (0.4)	0 (0.0)	1 (0.9)	
Obesity class 3	1 (0.4)	0 (0.0)	1 (0.9)	
Proteinuria > 300 mg/dL	2 (0.8)	0 (0.0)	2 (1.6)	<0.001
Microscopic hematuria	10 (4.2)	2 (1.7)	8 (6.5)	<0.001
Relevant alterations during protocol				<0.001
Metabolic syndrome	10 (4.2)	0 (0.0)	10 (8.1)	
SAH	6 (2.5)	0 (0.0)	6 (4.8)	
Nephrolithiasis	5 (2.1)	0 (0.0)	5 (4.0)	
Non-specific urine alterations	4 (1.7)	2 (1.7)	2 (1.6)	
Diabetes mellitus	4 (1.7)	0 (0.0)	4 (3.2)	
Chronic kidney disease	4 (1.7)	0 (0.0)	4 (3.2)	
Factor VII alterations	1 (0.8)	0 (0.0)	1 (0.8)	
ADPKD	1 (0.4)	0 (0.0)	1 (0.8)	
Polycystic ovary syndrome	1 (0.4)	0 (0.0)	1 (0.8)	
Complex renal cysts	1 (0.4)	0 (0.0)	1 (0.8)	
Uncontrolled hypothyroidism	1 (0.4)	0 (0.0)	1 (0.8)	
Impaired fasting glucose	1 (0.4)	0 (0.0)	1 (0.8)	
Mayer – Rokitansky syndrome	1 (0.4)	0 (0.0)	1 (0.8)	
Hepatic steatosis	1 (0.4)	0 (0.0)	1 (0.8)	
Fibromuscular dysplasia	1 (0.4)	0 (0.0)	1 (0.8)	
Relevant abnormalities in diagnostic test detected in protocol	17 (8.0)	1 (0.9)	16 (7.5)	<0.001
Nephrolithiasis	5 (2.1)	0 (0.0)	5 (4.0)	
Complex renal cysts	3 (1.3)	0 (0.0)	3 (2.4)	
CKD (chronic changes)	2 (0.8)	0 (0.0)	2 (1.6)	
Renal artery stenosis	1 (0.4)	0 (0.0)	1 (0.8)	
Right renal atrophy	1 (0.4)	0 (0.0)	1 (0.8)	
Uterine hypoplasia and mullerian malformations	1 (0.4)	0 (0.0)	1 (0.8)	
Hepatomegaly in test	1 (0.4)	1 (0.9)	0 (0.0)	
ADPKD	1 (0.4)	0 (0.0)	1 (0.8)	
Malignant ovarian cysts	1 (0.4)	0 (0.0)	1 (0.8)	
Pyelocaliceal ectasia	1 (0.4)	0 (0.0)	1 (0.8)	
Psychological disorder that contraindicates the KT	4 (1.7)	0 (0.0)	4 (3.2)	<0.001
Detected infections in protocol				<0.001
Latent tuberculosis	13 (5.4)	6 (5.2)	7 (5.6)	
Hepatitis A virus	1 (0.4)	0 (0.0)	1 (0.8)	
Glomerular Filtration Rate (mL/min/1.73 m^2^)				0.005
> 90.0	196 (81.6)	103 (88.8)	93 (75.0)	
80.0–89.9	24 (10.0)	10 (8.6)	14 (11.3)	
66.4–79.9	20 (8.4)	3 (2.6)	17 (13.7)	

Abbreviations – n: number; HIV: Human immunodeficiency virus; mg: milligrams; dL: deciliter; SAH: Systemic Arterial Hypertension; Alt: alterations; ADPKD: Autosomal dominant polycystic kidney disease; CKD: Chronic Kidney Disease; KT: Kidney transplant; ml milliliters; min: minutes; m: meter.

A risk factor analysis was conducted using bivariate logistic regression to evaluate kidney transplant protocols. Recipient age from 18 years was found to be statistically significant; for each additional year of recipient age, the risk increases by 3.1%, corresponding to an odds ratio (OR) of 1.031 (95% confidence interval [CI]: 1.006–1.056, p = 0.014). The determination of PRA CI showed an OR of 5.389 (95% CI: 2.124–13.671, p < 0.001). For donors, risk factors included structural alterations detected in cabinet tests (OR: 23.234, 95% CI: 5.263–102.561, p < 0.001), a GFR between 80.0 and 89.9 mL/min/1.73 m^2^ by CKD-EPI (OR: 6.276, 95% CI: 1.782–22.103, p = 0.012), and a history of infections (OR: 9.056, 95% CI: 3.671–23.338, p < 0.001). Results are summarized in Table 3.

**Table 3:** Risk factors for not completing a kidney transplant protocol

Variable	OR	95% Confidence interval		p
		Upper	Lower	
Receptor-related variables				
Age (years)	1.031	1.006	1.056	0.014
Chronic degenerative diseases (Without reference)				
DM1, DM2, DM-SAH	1.925	0.516	7.177	0.329
SAH	1.46	0.448	4.763	0.530
Panel Reactive Antibody				
Class I (more than 30%)	5.389	2.124	13.671	<0.001
Class II (more than 30%)	1.481	0.588	3.731	0.405
Donor-specific antibody	1.564	0.815	3.001	0.178
Active infections	1.264	0.425	3.761	0.673
Anemia	1.532	0.813	2.886	0.187
Abnormal transthoracic echocardiogram	1.463	0.773	2.77	0.242
Cardiovascular extension test	1.946	0.908	4.171	0.087
Donor-related variables				
Age (years)	1.005	0.983	1.028	0.640
Overweight/Obesity	0.796	0.436	1.455	0.458
GFR (mL/min/1.73 m^2^)	0.484	0.122	2.255	0.386
> 90.0 (Ref.)				
80.0–89.9	6.276	1.782	22.103	0.004
66.4–79.9	1.551	0.657	3.659	0.317
Documented pathologies				
Microscopic Hematuria	4.75	0.985	22.902	0.052
Abnormalities in diagnostic test	23.234	5.263	102.56	<0.001
Detected infections in protocol	9.056	3.671	22.338	<0.001

Abbreviations – OR: odds ratio; ref.: reference; DM: diabetes mellitus; SAH: Systemic Arterial Hypertension; ml: milliliters; min: minutes; m: meter; KT: kidney transplant; GFR: Glomerular Filtration Rate.

**Figure 1:**
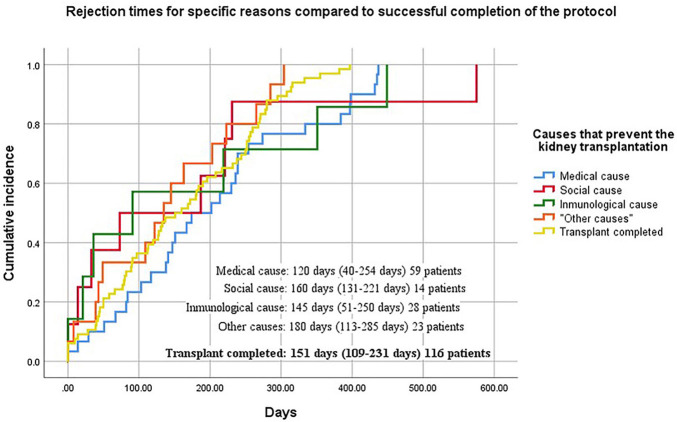
Rejection times for specific reasons compared to successful completion of the protocol.

The average time for acceptance or rejection of a protocol was 151 days, with an interquartile range (IQR 25–75) of 115–186 days. Patients who did not complete the protocol were classified into 4 groups: 1) rejection due to medical causes, 59 potential recipients (24.6%), with a rejection time of 120 days (IQR 40–254 days); 2) 14 individuals (5.8%) did not complete the protocol due to psychosocial causes, with a median time of 160 days (IQR 131-221 days); 3) rejection due to immunological causes, 28 subjects (11.7%), with a median rejection time of 145 days (IQR 51–250 days); and 4) rejection due to other causes, 23 subjects (9.6%), with a median of 180 days (IQR 113–285 days). See Figure 1 and Table 4. Of the 124 (51.6%) discarded donor-recipient pairs, 80 (33.3%) were due to donor-related causes and 44 (18.3%) were due to recipient-related causes.

**Table 4:** Kidney transplant time

Variable	n (%)	Estimated time (days) for the conclusion/rejection of protocol		
		25%	50%	75%
Concluded transplant	116 (48.3)	109	151	231
Did not conclude transplant (n = 124)				
Medical	59 (24.6)	40	120	254
Psychosocial	14 (5.8)	131	160	221
Immunological	28 (11.7)	51	145	250
Others	23 (9.6)	113	180	285

Abbreviation – n: number.

## Discussion

### Main Findings

In our protocol, 240 donor-recipient pairs were evaluated. Of these, 116 (48.3%) completed transplantation, while 124 (51.7%) did not proceed to transplant. Only 89 pairs (37.1%) completed the evaluation protocol within 18 weeks; of these, 38 (15.8%) received a kidney transplant and 51 (21.3%) were excluded. The remaining 151 pairs (62.9%) required more than 18 weeks to complete evaluation; among these, 78 (32.5% of the total cohort) were transplanted and 73 (30.4%) were excluded. The principal reasons for exclusion at our center were loss of adherence to the protocol, a high degree of sensitization in recipients, and positive crossmatch results prior to transplantation.

### Comparison

Habbous et al.^6^ reported median ages of 45 years for recipients and 44 years for donors. In contrast, the median ages in our cohort were substantially lower: 33 years for potential recipients and 35 years for potential donors. This disparity may reflect incomplete case finding in our population and suggests a need to intensify early detection of CKD so that disease progression can be tracked and the proportion of patients reaching renal replacement therapy can be reduced. Recipient sensitization in our series was higher in individuals with prior pregnancies; Paige M. Porett^18^ reported that two pregnancies may increase the risk of sensitization to as high as 62%. Donor estimated glomerular filtration rate (eGFR) was an important determinant of transplant success. Donors with eGFR <90 mL/min/1.73 m^2^ were more likely to be excluded from the protocol. Habbous et al.^6^ found higher transplant rates when the donor’s eGFR was ≥90 mL/min/1.73 m^2^.

The median time to accept or reject a living-donor transplant protocol at our center was 151 days. Although this interval is shorter than the duration reported by some centers (up to 10 months), it remains longer than the median reported by the Oxford Transplant Centre (132 days)^19^. National guidelines in the United Kingdom and Ireland recommend completion of the transplant protocol within 18 weeks from initial evaluation to surgery.

Multiple barriers prevented completion of the protocol. Medical contraindications in potential donors included nephrolithiasis, fibromuscular dysplasia, and vascular disease. Lorenz et al.^15^ reported similar findings in 1,957 potential donors: nephrolithiasis (11%), fibromuscular dysplasia (2.8%), and renal artery atherosclerosis or stenosis (5.3%). The 2017 KDIGO guidelines recommend a comprehensive approach to donor evaluation rather than decisions based on isolated risk factors^20^.

### Psychosocial and Economic Barriers were also Significant

Concerns about loss of income during recovery, fear of surgery, inadequate counseling about postoperative care, and anxiety about losing an organ. These factors can reduce adherence and increase refusal rates. Interventions such as perioperative counseling, psychosocial support and financial protection (for example, paid sick leave) may mitigate these barriers. In our center, we provide paid disability leave for one month and follow-up care for donors for one year, after which care is transferred to their primary care unit^21,22^.

Immunologic sensitization was a major impediment to transplantation. Crossmatch testing identified 15 positive cases (6.3% of the cohort). Twenty-four patients (10.0%) had PRA >30% for class I, and 22 patients (9.2%) had PRA >30% for class II; only 8 (3.3% of the cohort) and 13 (5.4% of the cohort), respectively, underwent transplantation. Donor-specific antibody (DSA) testing was performed in 169 patients (70.4% of the cohort), and DSA were detected in 97 patients (40.4% of the total cohort). Of those with DSA, 59 (24.6% of the cohort) completed transplantation, while 38 (15.8%) were excluded. Many patients with high-MFI DSA were excluded without additional testing (such as C1q complement fixation, Ig subclass typing, or cytotoxic crossmatch) that could have clarified transplant eligibility or suitability for desensitization. The high direct cost of these additional assays and desensitization therapies limits their availability in many public institutions^10,23^.

Structural abnormalities leading to donor exclusion included nephrolithiasis in 5 cases (2.1%), complex renal cysts in 3 (1.3%), chronic structural renal alterations in 2 (0.8%), autosomal dominant polycystic kidney disease in 1 (0.4%), and renal artery stenosis in 1 (0.4%). These findings are consistent with Perlis et al.^24^, who reported nephrolithiasis as one of the leading causes of donor exclusion.

### Implications

Strategies to improve donor acceptance and completion of the transplant pathway include enhanced education on self-care and coping with nephrectomy, structured psychological counseling to address grief and loss related to donation, and clear information about postoperative recovery and long-term follow up. Improving socioeconomic support (for example, paid leave and transportation subsidies), translating educational materials into indigenous languages, and optimizing referral networks to reduce travel burden could reduce attrition, particularly in underserved populations.

### Limitations

This was a single-center, retrospective, descriptive study; therefore, the findings are subject to selection bias, limited generalizability, a relatively small sample size, incomplete clinical data in some records, and a short follow-up period^22^. The absence of routine advanced immunologic testing (C1q, Ig subclass, cytotoxic crossmatch) and the limited availability of desensitization protocols in the public system constrained management options for highly sensitized patients. Addressing these limitations in future multicenter or prospective studies would increase statistical power and improve the external validity of our observations.

## Conclusions

Medical causes were the leading barriers to completing living donor kidney transplant protocols, followed by immunologic and psychosocial factors. Streamlined evaluations, early crossmatching, and psychosocial support could improve completion rates.

## Data Availability

The datasets generated and/or analyzed during the current study are not publicly available due to ethical/legal/privacy restrictions but are available from the corresponding author upon reasonable request.
