# Supplementary material for: Factors that preclude the conclusion of a kidney transplant protocol from a living donor
Source: J Bras Nefrol. 2026 Apr 17;48(2):e20240157. doi: 10.1590/2175-8239-JBN-2024-0157en (PMC13123570; doi:10.1590/2175-8239-JBN-2024-0157en)
Supplement: Translation

# Translation: Fatores que impedem a conclusão do protocolo de transplante renal com doador vivo

**DOI:** 10.1590/2175-8239-JBN-2024-0157pt

**Authors:** Alfonso Rodríguez Ojeda, Raúl Edgar Santacruz Adi, Nelly Gonzalez Audiffred, Rogelio Iván Silva Rueda, Fabiola Pazos Pérez, Maria Inés Gil Arredondo, Javier Rivera Flores, Jorge Velez Silva, Adriana Flores Palacios, Juan Carlos H. Hernández Rivera

**Affiliations:** 1 Instituto Mexicano del Seguro Social, Centro Médico Nacional Siglo XXI, Hospital de Especialidades, Servicio de Nefrología, Ciudad de México, México.; 2 Instituto Mexicano del Seguro Social, Centro Médico Nacional Siglo XXI, Hospital de Especialidades, Unidad de Investigación Médica en Enfermedades Nefrológicas, Ciudad de México, México.

**Keywords:** Protocolo de Transplante, Doadores Vivos, Rejeição de Enxerto

## Abstract

**Introdução:**

O transplante renal (TR) é uma terapia renal substitutiva que proporciona maior sobrevida e melhor qualidade de vida; no entanto, nem sempre é bem-sucedido devido a fatores sociais, físicos, clínicos, imunológicos ou à falta de adesão.

**Métodos:**

Este estudo retrospectivo, analítico e longitudinal incluiu pacientes inseridos em protocolo de TR com doador vivo entre maio de 2021 e novembro de 2022. Foram avaliados os fatores que impediram a conclusão desse protocolo. Análises comparativas e curvas de incidência cumulativa foram utilizadas para avaliar o tempo até a conclusão ou a descontinuação do protocolo. A análise foi realizada utilizando o IBM SPSS Statistics, versão 26 (IBM Corp., EUA).

**Resultados:**

Entre 240 pares doador-receptor, 116 (48,3%) concluíram com sucesso o TR, enquanto 124 (51,6%) não o completaram. As razões para a não conclusão incluíram causas clínicas (59/124; 47,6%), incompatibilidade imunológica (28/124; 22,6%), fatores psicossociais (14/124; 11,3%) e outras causas (23/124; 18,5%). O tempo mediano até a aceitação ou descontinuação do protocolo foi de 151 dias (IIQ 115–186). Entre os indivíduos que efetivamente foram submetidos ao TR, o tempo mediano de conclusão também foi de 151 dias (IIQ 109–231).

**Conclusões:**

As condições clínicas constituíram as principais barreiras à conclusão dos protocolos de TR com doador vivo, seguidas por fatores imunológicos e psicossociais. A otimização das avaliações médicas, a implementação de triagem imunológica precoce e o reforço do suporte psicossocial podem contribuir para o aumento das taxas de conclusão desses protocolos.

## Introdução

Estima-se que entre 8% e 15% da população mundial seja acometida por doença renal crônica (DRC). Nos países de renda média, as doenças crônicas não transmissíveis representam aproximadamente 71% da carga total de doenças, e a doença renal em estágio terminal (DRET) representa cerca de 15% dos gastos anuais em saúde^1,2^.

Nem todos os pacientes com DRC são candidatos a transplante renal (TR). Em comparação com o transplante de doador falecido, o TR com doador vivo oferece diversas vantagens, incluindo maior sobrevida do paciente e do enxerto e menor tempo de espera até o transplante^3,4,5,6^. Pacientes em hemodiálise (HD) ou diálise peritoneal (DP) geralmente apresentam um agravamento progressivo do estado geral de saúde, da situação profissional, do bem-estar emocional, dos níveis de energia, da função sexual e da qualidade do sono^7,8^. Em 2022, no México, aproximadamente 7 em cada 10 TR foram realizados a partir de doadores vivos. As baixas taxas de transplante em países de baixa e média renda são atribuíveis principalmente a deficiências na infraestrutura de saúde. Os desfechos dos transplantes, particularmente a sobrevida a médio e longo prazos, dependem do acesso a medicamentos imunossupressores, do estado nutricional e do controle de doenças infecciosas. Desigualdades sociais, culturais e econômicas, juntamente com múltiplos fatores imunológicos e não imunológicos, influenciam tanto o acesso ao transplante quanto os desfechos pós-transplante^9^.

A agilidade na avaliação do doador vivo é fundamental, tendo como objetivos a redução da comorbidade e a diminuição dos custos para os pacientes em terapia renal substitutiva. Artigos de revisão relatam variabilidade nos prazos de avaliação do doador, com intervalos-alvo de aproximadamente 18 semanas descritos nos Estados Unidos e no Reino Unido^10–12^. Em uma coorte canadense de 887 doadores, o tempo mediano entre o encaminhamento e a doação foi de 10,5 meses (IIQ 6,9–17,7 meses); notavelmente, 122 dos 360 pacientes em lista de espera para transplante precisaram iniciar terapia renal substitutiva devido a atrasos^13^. Um estudo norte-americano relatou tempo mediano de 9,2 meses, com doadores afro-americanos apresentando um intervalo mediano ligeiramente mais longo (10,2 meses) em comparação aos não afro-americanos (8,9 meses). O processo de avaliação é comumente dividido em cinco etapas^1^: encaminhamento^2^, triagem médica^3^, avaliação^4^, liberação^5^ e doação^14^. Uma revisão sistemática sugere um prazo mediano típico de aproximadamente 10 meses; até 25% dos protocolos excedem 16 meses, muitas vezes devido a investigações adicionais, necessidade de perda de peso ou intervenções para abstinência de substâncias que podem adicionar de 3 a 9 meses ao processo^6,15,16^.

Diversos fatores podem impedir a conclusão do TR com doador vivo; essas deliberações priorizam a segurança tanto do doador quanto do receptor^17^. Este estudo teve como objetivo identificar os fatores associados à não conclusão dos protocolos de transplante renal com doador vivo, bem como os tempos médios de cada grupo designado operacionalmente.

## Métodos

Este foi um estudo retrospectivo, analítico e longitudinal, realizado entre 1º de maio de 2021 e 30 de novembro de 2022. Foram analisados todos os casos disponíveis. O estudo incluiu pacientes com DRC estágios 4–5 da KDIGO (*Kidney Disease: Improving Global Outcomes*), encaminhados como pares doador-receptor, de qualquer sexo e com idade superior a 18 anos. Considerou-se critério de exclusão a ausência de dados epidemiológicos necessários nos prontuários clínicos; entretanto, nenhum par doador-receptor foi, por fim, excluído. O estudo foi aprovado pelo Comitê Local de Pesquisa e Ética em Saúde do IMSS, número de aprovação 3601, com registro institucional número R-2023-3601-050, datado de 3 de fevereiro de 2023.

Os dados de cada par doador-receptor, incluindo as avaliações de cada especialista envolvido (cardiologista, urologista, cirurgião bucomaxilofacial, nefrologista, psiquiatra, nutricionista, clínico geral, etc.), bem como os exames bioquímicos e clínicos, foram analisados nos prontuários eletrônicos e físicos da instituição, mediante autorização prévia do Comitê de Ética e Pesquisa.

As razões para a não conclusão do protocolo e, consequentemente, para a não realização do transplante foram agrupadas operacionalmente em quatro categorias: 1) Clínicas: hipertensão arterial, proteinúria, litíase, atrofia renal, estenose arterial, doença policística, malignidade ou suspeita de malignidade, ectasia, infecções ativas, alterações cardíacas, obesidade mórbida, anormalidades no sedimento urinário, síndrome metabólica, diabetes mellitus, etc.; 2) Psicossociais: conforme determinação de avaliação psiquiátrica–como demência–ou avaliação psicológica–como ausência de uma rede adequada de suporte familiar; 3) Imunológicas: prova cruzada persistentemente positiva por diferentes métodos, incluindo CDC (Citotoxicidade Dependente do Complemento), painel de reatividade de anticorpos altamente sensibilizado ou presença de anticorpos específicos contra o doador em títulos elevados; e 4) Outras: para os fins desta investigação, incluíram-se nessa categoria causas que não puderam ser classificadas como clínicas, psicossociais (conforme determinado por um psiquiatra ou psicólogo) ou relacionadas à histocompatibilidade. Exemplos incluem a hesitação do doador em ceder um rim, a decisão do receptor de interromper o protocolo de transplante, óbito do receptor, mudança de residência para outro estado ou região, ou perda da cobertura pelo seguro social.

Para variáveis quantitativas, a média e o desvio padrão ou a mediana e o intervalo interquartil (IIQ 25–75) foram relatados de acordo com a distribuição dos dados. Variáveis categóricas foram descritas por meio de frequências e porcentagens. Foram realizadas comparações de grupo entre pacientes que concluíram com sucesso o protocolo de transplante renal e aqueles que não o completaram. O teste t de *Student* foi utilizado para analisar as diferenças entre as médias, o teste U de *Mann-Whitney* para as medianas e o teste exato de *Fisher* ou o teste qui-quadrado de *Pearson* para as variáveis qualitativas, conforme apropriado.

Foram construídas curvas de incidência cumulativa a partir do início do protocolo, concluídas quando os transplantes foram realizados ou quando o protocolo não foi concluído por qualquer motivo. Para todas as análises estatísticas descritas, considerou-se estatisticamente significativo um valor de p < 0,05. Todas as análises estatísticas foram realizadas utilizando o *IBM SPSS Statistics*, versão 26 (IBM Corp., Armonk, NY, EUA).

## Resultados

Foram incluídos 240 pares doador-receptor para fins de transplante. Entre os potenciais receptores, 126 eram homens (52,5%) e 114 eram mulheres (47,5%), sem diferenças estatísticas. Para a variável relacionada a potenciais doadores, a taxa de filtração glomerular apresentou significância estatística com valor de p = 0,002 no teste U de *Mann-Whitney* (Tabela 1).

**Tabela 1:** Características basais de potenciais receptores e potenciais doadores antes do protocolo

Variável	Total (n = 240)	Protocolo concluído (n = 116)	Protocolo não concluído (n = 124)	p
Variáveis dos potenciais receptores renais				
Sexo				0,115
Masculino	126 (52,5)	67 (57,8)	59 (47,6)	
Feminino	114 (47,5)	49 (42,2)	65 (52,4)	
Idade (anos)*	33 (28–42)	33 (28–39)	34 (27–48)	0,012
Doenças crônicas				0,605
DM 1	8 (3,3)	4 (3,4)	4 (3,2)	
DM 2	2 (0,8)	0 (0,0)	2 (1,6)	
HAS	190 (79,2)	93 (80,2)	97 (78,2)	
DM-HAS	28 (11,7)	12 (10,3)	16 (12,9)	
Tipo sanguíneo				0,112
O	166 (69,2)	82 (70,7)	84 (67,7)	
A	60 (25,0)	24 (20,7)	36 (29,0)	
B	11 (4,6)	7 (6,0)	4 (3,2)
AB	3 (1,3)	3 (2,6)	0 (0,0)	
Modalidade de TRS anterior ao TR				0,229
Hemodiálise	100 (41,7)	42 (36,2)	58 (46,8)	
Diálise peritoneal	100 (41,7)	55 (47,4)	45 (36,3)	
TR	1 (0,4)	0 (0,0)	1 (0,8)	
Preemptivo	39 (16,3)	19 (16,4)	20 (16,1)	
Etiologia da DRC				0,26
Indeterminada	158 (65,8)	80 (69,6)	78 (62,9)	
DM	24 (10,0)	10 (8,7)	14 (11,3)	
DRPAD	14 (5,8)	5 (4,3)	9 (7,3)	
GE focal e segmentar	8 (3,3)	6 (5,2)	2 (1,6)	
Refluxo vesicoureteral	7 (2,9)	5 (4,3)	2 (1,6)	
Nefrite lúpica	7 (2,9)	4 (3,5)	3 (2,4)	
GE MP	4 (1,7)	0 (0,0)	4 (3,2)	
HAS	3 (1,3)	2 (1,7)	1 (0,8)	
Vasculite ANCA	3 (1,3)	0 (0,0)	3 (2,4)	
Nefropatia por IgA	2 (0,8)	1 (0,9)	1 (0,8)	
Nefrite tubulointersticial	2 (0,8)	0 (0,0)	2 (1,6)	
Hiperuricemia	2 (0,8)	1 (0,9)	1 (0,8)	
Nefrolitíase	1 (0,4)	0 (0,0)	1 (0,8)	
Nefroangiosclerose	1 (0,4)	0 (0,0)	1 (0,8)	
Arterite de Takayasu	1 (0,4)	0 (0,0)	1 (0,8)	
Bexiga neurogênica	1 (0,4)	1 (0,9)	0 (0,0)	
Nefropatia por radiação	1 (0,4)	0 (0,0)	1 (0,8)	
Câncer renal	1 (0,4)	0 (0,0)	1 (0,8)	
Variáveis dos potenciais doadores renais				
Sexo		0,115
Masculino	93 (38,8)	39 (33,6)	54 (43,5)	
Feminino	147 (61,3)	77 (66,4)	70 (56,5)	
Idade (anos)*	35 (28–46)	35 (30–44)	36 (27–48)	0,641
Creatinina (mg/dL)*	0,77 (0,70–0,80)	0,77 (0,70–0,85)	0,78 (0,67–0,87)	0,111
TFG (mL/min/1,72 m^2^ ASC)*	109,0 (99,4–119,7)	109,0 (99,4–119,7)	108,1 (88,3–125,6)	0,002
Tipo sanguíneo				0,617
O	188 (78,3)	92 (79,3)	96 (77,4)	
A	42 (17,5)	18 (15,5)	24 (19,4)	
B	9 (3,8)	5 (4,3)	4 (3,2)	
AB	1 (0,4)	1 (0,9)	0 (0,0)	
Relação				0,346
Primo(a)	12 (5)	5 (4,3)	7 (5,6)	
Mãe/Pai	42 (17,5)	19 (16,4)	23 (18,5)	
Irmão/Irmã	86 (35,8)	45 (38,8)	41 (33,1)	
Esposo/Esposa	45 (18,8)	27 (23,3)	18 (14,5)	
Filho(a)	19 (7,9)	4 (3,4)	15 (12,1)	
Cunhado(a)	3 (1,3)	1 (0,9)	2 (1,6)	
Tio/Tia	9 (3,8)	4 (3,4)	5 (4,0)	
Avô/Avó	1 (0,4)	0 (0,0)	1 (0,9)	
Sobrinho(a)	2 (0,8)	1 (0,9)	1 (0,8)	
Nenhuma	21 (8,8)	10 (8,6)	11 (8,9)	

Abreviaturas – n: número; DM1: diabetes mellitus tipo 1; DM2: diabetes mellitus tipo 2; HAS: hipertensão arterial sistêmica; TRS: terapia renal substitutiva; TR: transplante renal; DRC: doença renal crônica; DRPAD: doença renal policística autossômica dominante; GE: glomeruloesclerose; MP: membranoproliferativa; ANCA: anticorpos anticitoplasma de neutrófilos; Ig: imunoglobulinas; mg: miligramas; min: minuto; mL: mililitro; m: metro; ASC: área de superfície corporal.

Notas – (*) Dados expressos como mediana e intervalo interquartil de 25–75. A comparação entre grupos foi realizada utilizando o teste U de Mann-Whitney.

Entre as características do protocolo, algumas variáveis estatisticamente significativas foram observadas nos potenciais receptores renais. A prova cruzada (*crossmatch*) foi positiva em 15 casos (6,3%), em que todos completaram o protocolo (qui-quadrado; p < 0,001). O Painel de Reatividade de Anticorpos (PRA) classe I > 30% foi encontrado em 24 casos (10%): 8 casos (7,8%) entre aqueles que concluíram o protocolo e 16 casos (30,8%) entre aqueles que não o concluíram, com diferença estatisticamente significativa entre os grupos (qui-quadrado; p < 0,001). Para as variáveis relacionadas aos potenciais doadores, foram encontradas diferenças estatisticamente significativas entre os grupos (qui-quadrado; p < 0,001) para proteinúria superior a 300 mg/dL, hematúria microscópica, alterações clínicas ou detectadas em consultório durante o protocolo, distúrbios psicológicos em quatro casos e infecções ocorridas no decorrer do processo. Para a função renal, a taxa de filtração glomerular foi analisada em três grupos, evidenciando uma diferença significativa entre os grupos (qui-quadrado; p = 0,005) (Tabela 2).

**Tabela 2:** Características dos potenciais receptores e potenciais doadores renais durante o protocolo

Variável	Total (n = 240)	Protocolo concluído (n = 116)	Protocolo não concluído (n = 124)	p
Variáveis dos potenciais receptores renais			
Teste de prova cruzada positivo	15 (6,3)	0 (0,0)	15 (78,9)	<0,001
Painel de Reatividade de Anticorpos			
Classe I (> 30%)	24 (10,0)	8 (7,6)	16 (30,8)	<0,001
Classe II (> 30%)	22 (9,2)	13 (12,4)	9 (17,3)	0,403
Anticorpo específico contra o doador	97 (40,4)	59 (53,6)	38 (64,4)	0,177
Infecções ativas	11 (4,9)	3 (2,6)	8 (7,3)	0,105
Abscesso renal	1 (0,4)	0 (0,0)	1 (0,9)
Peritonite	1 (0,4)	0 (0,0)	1 (0,9)
Pneumonia	2 (0,8)	1 (0,9)	1 (0,9)	
HIV	1 (0,4)	0 (0,0)	1 (0,9)	
Tuberculose latente	6 (2,5)	2 (1,7)	4 (3,6)	
Anemia	190 (79,2)	96 (82,8)	94 (75,8)	0,185
Transtorno psicológico	1 (0,4)	0 (0,0)	1 (0,9)	0,297
Ecocardiograma transtorácico alterado	171 (71,3)	93 (80,9)	78 (74,3)	0,241
Teste de esforço cardiovascular alterado	34 (14,2)	14 (15,1)	20 (25,6)	0,084
Variáveis dos potenciais doadores renais			
Alterações do peso corporal				0,234
Baixo peso	55 (24,4)	29 (25,7)	26 (23,2)	
Sobrepeso	124 (55,1)	65 (57,5)	59 (52,7)	
Obesidade grau 1	40 (17,8)	16 (14,2)	24 (21,4)	
Obesidade grau 2	1 (0,4)	0 (0,0)	1 (0,9)	
Obesidade grau 3	1 (0,4)	0 (0,0)	1 (0,9)	
Proteinúria > 300 mg/dL	2 (0,8)	0 (0,0)	2 (1,6)	<0,001
Hematúria microscópica	10 (4,2)	2 (1,7)	8 (6,5)	<0,001
Alterações relevantes durante o protocolo				<0,001
Síndrome metabólica	10 (4,2)	0 (0,0)	10 (8,1)	
HAS	6 (2,5)	0 (0,0)	6 (4,8)	
Nefrolitíase	5 (2,1)	0 (0,0)	5 (4,0)	
Alterações urinárias não específicas	4 (1,7)	2 (1,7)	2 (1,6)	
Diabetes mellitus	4 (1,7)	0 (0,0)	4 (3,2)	
Doença renal crônica	4 (1,7)	0 (0,0)	4 (3,2)	
Deficiência do fator VII	1 (0,8)	0 (0,0)	1 (0,8)	
DRPAD	1 (0,4)	0 (0,0)	1 (0,8)	
Síndrome dos ovários policísticos	1 (0,4)	0 (0,0)	1 (0,8)	
Cistos renais complexos	1 (0,4)	0 (0,0)	1 (0,8)	
Hipotireoidismo não controlado	1 (0,4)	0 (0,0)	1 (0,8)	
Glicemia de jejum alterada	1 (0,4)	0 (0,0)	1 (0,8)	
Síndrome de Mayer-Rokitansky	1 (0,4)	0 (0,0)	1 (0,8)	
Esteatose hepática	1 (0,4)	0 (0,0)	1 (0,8)	
Displasia fibromuscular	1 (0,4)	0 (0,0)	1 (0,8)	
Anomalias relevantes no teste diagnóstico detectadas durante o protocolo	17 (8,0)	1 (0,9)	16 (7,5)	<0,001
Nefrolitíase	5 (2,1)	0 (0,0)	5 (4,0)	
Cistos renais complexos	3 (1,3)	0 (0,0)	3 (2,4)	
DRC (alterações crônicas)	2 (0,8)	0 (0,0)	2 (1,6)	
Estenose da artéria renal	1 (0,4)	0 (0,0)	1 (0,8)	
Atrofia renal direita	1 (0,4)	0 (0,0)	1 (0,8)	
Hipoplasia uterina e malformações mullerianas	1 (0,4)	0 (0,0)	1 (0,8)	
Hepatomegalia em exame de imagem	1 (0,4)	1 (0,9)	0 (0,0)	
DRPAD	1 (0,4)	0 (0,0)	1 (0,8)	
Cistos ovarianos malignos	1 (0,4)	0 (0,0)	1 (0,8)	
Ectasia pielocalicial	1 (0,4)	0 (0,0)	1 (0,8)	
Distúrbio psicológico que contraindica o TR	4 (1,7)	0 (0,0)	4 (3,2)	<0,001
Infecções detectadas durante o protocolo				<0,001
Tuberculose latente	13 (5,4)	6 (5,2)	7 (5,6)	
Vírus da hepatite A	1 (0,4)	0 (0,0)	1 (0,8)	
Taxa de filtração glomerular (mL/min/1,73 m^2^)				0,005
> 90,0	196 (81,6)	103 (88,8)	93 (75,0)	
80,0–89,9	24 (10,0)	10 (8,6)	14 (11,3)	
66,4–79,9	20 (8,4)	3 (2,6)	17 (13,7)	

Abreviaturas – n: número; HIV: vírus da imunodeficiência humana; mg: miligramas; dL: decilitro; HAS: hipertensão arterial sistêmica; Alt: alterações; DRPAD: doença renal policística autossômica dominante; DRC: doença renal crônica; TR: transplante renal; ml: mililitros; min: minutos; m: metro.

Foi realizada uma análise de fatores de risco utilizando regressão logística bivariada para avaliação dos protocolos de transplante renal. A idade do receptor, a partir de 18 anos, mostrou-se estatisticamente significativa; para cada ano adicional na idade do receptor, o risco aumentou em 3,1%, correspondendo a um *odds ratio* (OR) de 1,031 (intervalo de confiança [IC] de 95%: 1,006–1,056; p = 0,014). A determinação do PRA CI apresentou OR de 5,389 (IC 95%: 2,124–13,671; p < 0,001). Para os doadores, os fatores de risco incluíram alterações estruturais detectadas em exames de consultório (*cabinet tests*) (OR: 23,234; IC 95%: 5,263–102,561; p < 0,001), uma TFG entre 80,0 e 89,9 mL/min/1,73 m^2^ pela equação CKD-EPI (OR: 6,276; IC 95%: 1,782–22,103; p = 0,012) e histórico de infecções (OR: 9,056; IC 95%: 3,671–23,338; p < 0,001). Os resultados estão resumidos na Tabela 3.

**Tabela 3:** Fatores de risco para a não conclusão do protocolo de transplante renal

Variável	OR	Intervalo de confiança de 95%		p
		Superior	Inferior	
Variáveis relacionadas ao receptor			
Idade (anos)	1,031	1,006	1,056	0,014
Doenças degenerativas crônicas (sem referência)				
DM1, DM2, DM-HAS	1,925	0,516	7,177	0,329
HAS	1,46	0,448	4,763	0,530
Painel de Reatividade de Anticorpos				
Classe I (> 30%)	5,389	2,124	13,671	<0,001
Classe II (> 30%)	1,481	0,588	3,731	0,405
Anticorpo específico contra o doador	1,564	0,815	3,001	0,178
Infecções ativas	1,264	0,425	3,761	0,673
Anemia	1,532	0,813	2,886	0,187
Ecocardiograma transtorácico alterado	1,463	0,773	2,77	0,242
Teste de estresse cardiovascular	1,946	0,908	4,171	0,087
Variáveis relacionadas ao doador				
Idade (anos)	1,005	0,983	1,028	0,640
Sobrepeso/Obesidade	0,796	0,436	1,455	0,458
TFG (mL/min/1,73 m^2^)	0,484	0,122	2,255	0,386
> 90,0 (Ref.)				
80,0–89,9	6,276	1,782	22,103	0,004
66,4–79,9	1,551	0,657	3,659	0,317
Patologias documentadas				
Hematúria microscópica	4,75	0,985	22,902	0,052
Anormalidades no teste diagnóstico	23,234	5,263	102,56	<0,001
Infecções detectadas durante o protocolo	9,056	3,671	22,338	<0,001

Abreviaturas – OR: odds ratio; ref.: referência; DM: diabetes mellitus; HAS: hipertensão arterial sistêmica; ml: mililitros; min: minutos; m: metro; TR: transplante renal; TFG: taxa de filtração glomerular.

O tempo médio para aceitação ou rejeição de um protocolo foi de 151 dias, com intervalo interquartil (IIQ 25–75) de 115–186 dias. Os pacientes que não concluíram o protocolo foram classificados em quatro grupos: 1) rejeição por causas clínicas: 59 potenciais receptores (24,6%), com tempo de rejeição de 120 dias (IIQ 40–254 dias); 2) causas psicossociais: 14 indivíduos (5,8%), com tempo mediano de 160 dias (IIQ 131–221 dias); 3) causas imunológicas, 28 indivíduos (11,7%), com tempo mediano de rejeição de 145 dias (IIQ 51–250 dias); e 4) outras causas: 23 indivíduos (9,6%), com tempo mediano de 180 dias (IIQ 113–285 dias). Ver Figura 1 e Tabela 4. Dos 124 (51,6%) pares doador-receptor descartados, 80 (33,3%) foram atribuídos a causas relacionadas ao doador e 44 (18,3%) a causas relacionadas ao receptor.

**Figura 1:** Tempos de rejeição por razões específicas em comparação com a conclusão bem-sucedida do protocolo.

**Tabela 4:** Tempo até o transplante renal

Variável	n (%)	Tempo estimado (em dias) para a conclusão/rejeição do protocolo		
		25%	50%	75%
Transplante concluído	116 (48,3)	109	151	231
Não concluíram o transplante (n = 124)				
Clínica	59 (24,6)	40	120	254
Psicossocial	14 (5,8)	131	160	221
Imunológica	28 (11,7)	51	145	250
Outras	23 (9,6)	113	180	285

Abreviatura – n: número.

## Discussão

### Principais Achados

Em nosso protocolo, foram avaliados 240 pares doador-receptor. Destes, 116 (48,3%) concluíram o transplante, enquanto 124 (51,7%) não deram continuidade ao procedimento. Apenas 89 pares (37,1%) completaram o protocolo de avaliação em até 18 semanas; desses, 38 (15,8%) receberam um transplante renal e 51 (21,3%) foram excluídos. Os 151 pares restantes (62,9%) necessitaram de mais de 18 semanas para concluir a avaliação; entre estes, 78 (32,5% da coorte total) foram transplantados e 73 (30,4%) foram excluídos. As principais razões para a exclusão em nosso centro foram a perda de adesão ao protocolo, um alto grau de sensibilização nos receptores e resultados positivos na prova cruzada antes do transplante.

### Comparação

Habbous et al.^6^ relataram idades medianas de 45 anos para os receptores e 44 anos para os doadores. Em contraste, em nossa coorte, as idades medianas foram substancialmente mais baixas: 33 anos para potenciais receptores e 35 anos para potenciais doadores. Essa disparidade pode refletir uma identificação incompleta de casos em nossa população e sugere a necessidade de intensificar a detecção precoce da DRC, para que a progressão da doença possa ser monitorada e a proporção de pacientes que evoluem para terapia renal substitutiva possa ser reduzida. A sensibilização dos receptores em nossa série foi maior em indivíduos com gestações prévias; Paige M. Porett^18^ relatou que duas gestações podem aumentar o risco de sensibilização em até 62%. A taxa de filtração glomerular estimada (TFGe) do doador foi um importante determinante do sucesso do transplante. Doadores com TFGe <90 mL/min/1,73 m^2^ apresentaram maior probabilidade de exclusão do protocolo. Habbous et al.^6^ observaram taxas de transplante mais elevadas quando a TFGe do doador era ≥90 mL/min/1,73 m^2^.

O tempo mediano para a aceitação ou rejeição de um protocolo de transplante de doador vivo em nosso centro foi de 151 dias. Embora esse intervalo seja inferior ao relatado por alguns centros (até 10 meses), ainda é superior à mediana relatada pelo *Oxford Transplant Centre* (132 dias)^19^. As diretrizes nacionais do Reino Unido e da Irlanda recomendam a conclusão do protocolo de transplante dentro de 18 semanas, desde a avaliação inicial até a realização da cirurgia.

Múltiplas barreiras impediram a conclusão do protocolo. As contraindicações clínicas em potenciais doadores incluíram nefrolitíase, displasia fibromuscular e doença vascular. Lorenz et al.^15^ relataram achados semelhantes em 1.957 potenciais doadores: nefrolitíase (11%), displasia fibromuscular (2,8%) e aterosclerose ou estenose da artéria renal (5,3%). As diretrizes KDIGO de 2017 recomendam uma abordagem abrangente para a avaliação do doador, em vez de decisões baseadas em fatores de risco isolados^20^.

### Barreiras Psicossociais e Econômicas Também Foram Significativas

Preocupações com a perda de renda no período de recuperação, medo da cirurgia, aconselhamento inadequado sobre cuidados pós-operatórios e ansiedade em relação à perda de um órgão. Esses fatores podem reduzir a adesão e aumentar as taxas de recusa. Intervenções como aconselhamento perioperatório, suporte psicossocial e proteção financeira (por exemplo, licença médica remunerada) podem mitigar essas barreiras. Em nosso centro, oferecemos licença por invalidez remunerada por um mês e cuidados de acompanhamento aos doadores pelo período de um ano, após o qual os cuidados são transferidos para sua unidade de atenção primária^21,22^.

A sensibilização imunológica constituiu um importante impedimento à realização do transplante. A prova cruzada identificou 15 casos positivos (6,3% da coorte). Vinte e quatro pacientes (10,0%) apresentaram PRA >30% para a classe I e 22 pacientes (9,2%) apresentaram PRA >30% para a classe II; apenas 8 (3,3% da coorte) e 13 (5,4% da coorte), respectivamente, foram submetidos ao transplante. O teste de anticorpos específicos contra o doador (DSA) foi realizado em 169 pacientes (70,4% da coorte), sendo detectados DSA em 97 pacientes (40,4% da coorte total). Entre aqueles com DSA, 59 (24,6% da coorte) concluíram o transplante, enquanto 38 (15,8%) foram excluídos. Muitos pacientes com DSA de alto MFI (*mean fluorescence intensity*) foram excluídos sem testes adicionais (como fixação do complemento C1q, tipagem de subclasse de Ig ou prova cruzada por citotoxicidade) que poderiam ter esclarecido a elegibilidade para o transplante ou a adequação para protocolos de dessensibilização. O elevado custo direto desses ensaios adicionais e terapias de dessensibilização limita sua disponibilidade em muitas instituições públicas^10,23^.

Alterações estruturais que levaram à exclusão de doadores incluíram nefrolitíase em 5 casos (2,1%), cistos renais complexos em 3 (1,3%), alterações renais estruturais crônicas em 2 (0,8%), doença renal policística autossômica dominante em 1 (0,4%) e estenose da artéria renal em 1 (0,4%). Esses achados são consistentes com os de Perlis et al.^24^, que descreveram a nefrolitíase como uma das principais causas de exclusão de doadores.

### Implicações

Estratégias para melhorar a aceitação de doadores e a conclusão do processo de transplante incluem educação aprimorada sobre autocuidado e enfrentamento da nefrectomia, aconselhamento psicológico estruturado para lidar com o luto e a perda relacionados à doação, e informações claras sobre a recuperação pós-operatória e o acompanhamento de longo prazo. O aprimoramento do suporte socioeconômico (por exemplo, licença remunerada e subsídios para transporte), a tradução de materiais educacionais para idiomas indígenas e a otimização das redes de encaminhamento para reduzir o ônus das viagens podem reduzir a evasão, especialmente em populações vulneráveis.

### Limitações

Este foi um estudo descritivo, retrospectivo e de centro único; portanto, os achados estão sujeitos a viés de seleção, generalização limitada, tamanho amostral relativamente pequeno, dados clínicos incompletos em alguns prontuários e um período de acompanhamento curto^22^. A ausência de testes imunológicos de rotina avançados (C1q, subclasse de Ig, prova cruzada por citotoxicidade) e a disponibilidade limitada de protocolos de dessensibilização no sistema público restringiram as opções de manejo para pacientes altamente sensibilizados. Abordar essas limitações em estudos futuros, multicêntricos ou prospectivos, poderia aumentar o poder estatístico e melhorar a validade externa de nossas observações.

## Conclusões

As causas clínicas constituíram as principais barreiras à conclusão dos protocolos de transplante renal com doador vivo, seguidas por fatores imunológicos e psicossociais. Avaliações otimizadas, a realização precoce de prova cruzada e o suporte psicossocial podem contribuir para a melhoria das taxas de conclusão do protocolo.

## Data Availability

Os conjuntos de dados gerados e/ou analisados durante o presente estudo não estão disponíveis publicamente devido a restrições éticas/legais/de privacidade, mas podem ser obtidos junto ao autor correspondente mediante solicitação razoável.
